# A New In Situ Coaxial Capacitive Sensor Network for Debris Monitoring of Lubricating Oil

**DOI:** 10.3390/s22051777

**Published:** 2022-02-24

**Authors:** Yishou Wang, Tingwei Lin, Diheng Wu, Ling Zhu, Xinlin Qing, Wendong Xue

**Affiliations:** School of Aerospace Engineering, Xiamen University, Xiamen 361005, China; wangys@xmu.edu.cn (Y.W.); tingweilin@163.com (T.L.); 35120181152309@stu.xmu.edu.cn (D.W.); 34720201151384@stu.xmu.edu.cn (L.Z.); xinlinqing@xmu.edu.cn (X.Q.)

**Keywords:** lubricant oil, online monitoring, capacitive sensor network, wear debris

## Abstract

Wear debris monitoring of lubricant oil is an important method to determine the health and failure mode of key components such as bearings and gears in rotatory machines. The permittivity of lubricant oil can be changed when the wear debris enters the oil. Capacitive sensing methods showed potential in monitoring debris in lubricant due to the simple structure and good response. In order to improve the detection sensitivity and reliability, this study proposes a new coaxial capacitive sensor network featured with parallel curved electrodes and non-parallel plane electrodes. As a kind of through-flow sensor, the proposed capacitive sensor network can be in situ integrated into the oil pipeline. The theoretical models of sensing mechanisms were established to figure out the relationship between the two types of capacitive sensors in the sensor network. The intensity distributions of the electric field in the coaxial capacitive sensor network are simulated to verify the theoretical analysis, and the effects of different debris sizes and debris numbers on the capacitance values were also simulated. Finally, the theoretical model and simulation results were experimentally validated to verify the feasibility of the proposed sensor network.

## 1. Introduction

The lubricating oil system is an important part of the aircraft engine. Besides lubrication and cooling, the lubricating oil can also be used as a transport medium of wear debris produced by mechanical wear. During normal operation, the concentration of wear particles in the lubricant oil remains constant, and the size of wear particles are small. When abnormal conditions occur, the concentration and size of the particles increase, which may cause malfunctioning of the machine. Monitoring the oil wear particles can not only indicate the health status of the oil system but it also can diagnose and evaluate the wear condition of the components [1].

At present, oil condition detection mainly includes offline and online methods. For offline methods, mainly including ferrography [2] and spectroscopy [3], the lubricating oil samples are collected and sent to laboratories for testing. Although offline methods provide more comprehensive information, both ferrography and spectroscopy require skilled operators and are time consuming.

Online oil debris detection mainly includes inductive detection [4], optical detection [5], ultrasonic detection [6], resistive detection [7], electrostatic charge detection [8], X-ray spectrometer [9] and capacitive detection [10,11]. Du et al. [12] have listed the advantages and disadvantages of all these online monitoring methods.

The most widely used inductive sensor is the MetalSCAN online debris monitoring sensor produced by Gastops [13]. Inductive sensors depend on the principle of magnetic induction; the magnetic field between the coils will be changed when metal debris passes through. This method can distinguish between ferromagnetic debris and non-ferromagnetic debris but cannot recognize non-metallic debris. The light transmission of oil is affected by the presence of debris thus debris contamination can be diagnosed by measuring light transmission [5]. However, the accuracy of the optical method is affected by the clarity of oil, the presence of bubbles and the properties of debris (refractivity, shape, etc.). Ultrasonic detection [6] is based on an acoustic wave scattering mechanism. When incident acoustic waves are scattered by abrasive particles, the amplitude of the wave reaching the receiver will decrease. The size of debris can be judged by the amplitude attenuation; however, the limitation of this method is that debris and bubbles cannot be distinguished. Resistive sensors can monitor ferrous debris by measuring oil resistance and make it easy to take a measurement. However, this method is difficult to determine oil contamination, and the results are affected by many factors. Electrostatic sensors detect oil debris by measuring the electrostatic charge carried by the debris [8]. The sensor is sensitive to small debris; however, it can only detect the oil containing a small amount of debris and cannot provide the correct information about the size and concentration of wear debris. X-ray spectroscopy is an online monitoring technology based on spectroscopic analysis [9], in which X-rays are used to excite and receive signals. This method requires complex analytical instruments and skilled operators, which is less feasible in practical applications.

**Figure 1:**
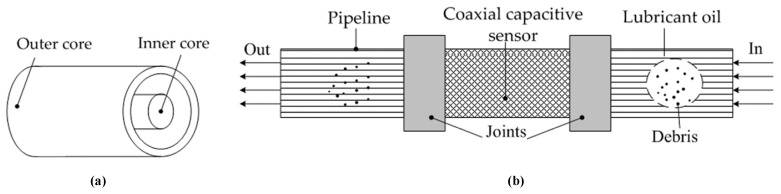
Schematic diagram of the presented capacitive sensor model: (**a**) Coaxial capacitive sensor model; (**b**) Integration scheme of the coaxial capacitive sensor and lubricant oil pipeline.

Capacitive-based methods can detect debris by monitoring the change in the permittivity of lubricating oil. These methods can simultaneously detect metallic debris and non-metallic debris and judge the size and concentration of abrasive particles according to the change of capacitance. Capacitive sensors are widely used due to their good temperature stability, simple structure, rapid dynamic response speed, strong adaptability and non-contact measurement. Although capacitive sensors are sensitive to the quality of lubricating oil, it is still the most practical and effective method. Murali et al. [14] proposed a microfluidic device based on the capacitive Coulter counting principle to detect debris by monitoring changes in the capacitance of two microelectrodes in a microfluidic channel. The device has high sensitivity and a low detection limit but also has a low throughput. Islam et al. [15] proposed a Thompson–Lampard theorem based cross-capacitive sensor to detect metal particles in lubricating oil. The sensor has four identical symmetrically placed electrodes, and the presence of metal debris in lubricant oil can be detected by monitoring changes in the cross-capacitance value. Muthuvel et al. [16] combined magnetic and capacitive features in a single sensor for debris monitoring. The proposed sensor plug uses a magnetic method to attract ferrous debris in lubricant oil, the presence of the debris accumulated on the plug can be sensed by a capacitive sensing technique. The sensor plug employs a differential method to make the sensor insensitive to parameters such as the variation in temperature and viscosity of oil, and the ferrous debris can also be collected by the sensor; however, the disadvantage of the sensor is that it cannot detect non-ferrous debris. Our team previously proposed a coaxial capacitive sensor and illustrated its characteristics through preliminary validation experiments [10,11,17]. Actually, in the previous work, ref. [10] investigated the changes in sensor performance under the influence of the temperature and flow rate. It was shown that with an increase in temperature, the measured capacitance will decrease since the dielectric permittivity of water or oil will increase with the temperature rise. As shown in Figure 1, the inner core and the outer core form two electrodes of the proposed coaxial capacitive sensor. The lubricant oil flows through the space between the outer core and inner core. The sensor can be in situ integrated with the lubricant pipe, which can measure ferrous debris, non-ferrous debris and other impurities at the same time. However, the sensitivity of this capacitive sensor is low due to the larger distance between two electrodes with different areas.

Based on the previous work of the authors, a coaxial capacitive sensor network is proposed to improve sensor performance. The novelty of the developed capacitive sensor network can be summarized in two aspects. First, compared with the original coaxial capacitive sensor, the inside space of the developed sensor is divided into multiple subspaces such that the distance between each pair of electrodes is reduced to improve the sensitivity on the premise of ensuring integration with the oil pipeline. Second, each subspace contains two pairs of different electrodes, which can obtain more information about wear debris according to different capacitive signals.

The main purpose of this paper is to analyze the feasibility of the proposed coaxial capacitive sensor network in oil debris monitoring. Firstly, the theoretical model of the coaxial capacitive sensor network is established. Next, the numerical simulation is carried out. Finally, the preliminary experiment is conducted, and the conclusions are drawn.

## 2. Design and Simulation of Coaxial Capacitive Sensor Network

### 2.1. Structure Design of Coaxial Capacitive Sensor Network

**Figure 2:**
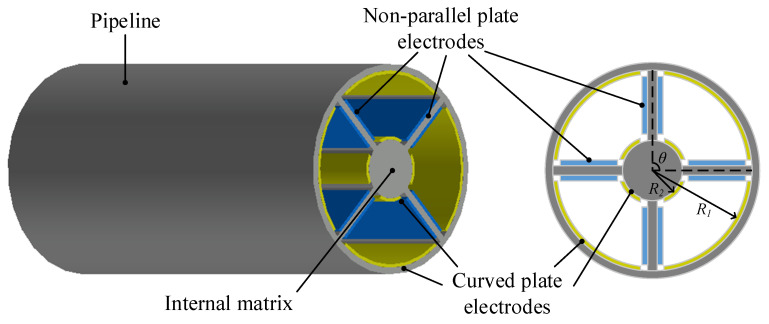
Model of coaxial capacitive sensor network.

In the authors’ opinion, the challenges of debris monitoring using flow-through sensors mainly derive from three aspects: (1) great size level variation between debris and lubricant moving space (i.e., lubricant pipeline); (2) the weak effect on relative physical and chemical parameters in the high noise level environment; (3) the dynamics of debris moving with lubricant oil. Inspired by the previous works and divide-and-conquer strategy, this study extended the coaxial capacitive sensor developed in the literature [11], creating a new coaxial capacitive sensor network, as shown in Figure 2. Without changing the structure of the oil pipeline, the oil flow region in the whole sensor was divided into four subspaces by the internal matrix; two pairs of different electrodes were arranged in each subspace, including non-parallel plate electrodes and curved plate electrodes. Therefore, the lubricating oil flowed through the four fan-shaped spaces among the pipeline and the matrix. The number of subspaces depends on the angle between a pair of non-parallel plate electrodes in the same space and the number of layers of parallel curved electrodes. In this paper, the interior of the sensor is divided into four subspaces, the included angle of the non-parallel plate electrode is 90°, and the number of layers of parallel curved electrodes is one. When the interior of the sensor is divided into multiple subspaces, the size level variation between the debris and lubricant pipeline is smaller than before, which solves the problem of the first challenge. At the same time, the four subspaces separated by the matrix are not connected to each other. When there is debris suspended in one area and no debris passes through other areas, the difference between the same types of capacitance in the different areas can be obtained to calculate the added capacitance caused by wear debris, and the influence of temperature, flow rate and other external factors can also be filtered out, which can solve the second problem. The shape of the debris is usually irregular; the effective area of the debris between the electrodes will continuously change when the debris flows with lubricant oil, resulting in a fluctuation of the measured capacitance. In the same detection area, different electrodes form different capacitances. According to different capacitance signals, more information regarding the abrasive particles can be obtained and the influence of the debris’ dynamics on the measured capacitance value is solved to a certain extent.

The manufacturing of the proposed sensor network must adapt to the requirements while improving the performance. In terms of material selection, the selection of electrode materials should meet the requirements of low corrosiveness, good conductivity and affordable prices. Since the proposed capacitive sensor network is directly in contact with the lubricating oil, the sensor electrodes should be designed to minimize the corrosive effect. Therefore, the pure metal electrodes of non-parallel and curved capacitive sensors are replaced with flexible composite thin films. As an alloy of copper and zinc, the brass is sputtered onto one side of the polyimide (PI) film. The PI film sides, with the brass as the conductive medium, are bonded on the internal matrix as the electrodes isolated from the lubricating oil by the PI film. The PI film has excellent corrosion resistance. In the real engineering application, the matrix to support the flexible PI film should be adapted to the operating temperature of the lubricating oil from −40 °C to +200 °C, and the selected material should have good electrical insulation properties and resistance to high temperature (up to +220 °C). Since the current study does not consider the temperature effect, PLA (polylactic acid) is selected as the material of the matrix made by a 3D printer. Finally, the electrodes are placed on the matrix, and the matrix is installed inside the sensor network.

### 2.2. Mathematical Model of the Capacitive Sensor Network

For traditional capacitive sensors [18], the capacitance (*C*) between the two parallel plates (electrodes) can be calculated according to Equation (1), where *ε_r_* is a relative dielectric constant depending on the dielectric material, *ε*_0_ is the constant of dielectric permissiveness in the vacuum, “*A*” is the plate’s common area and “*d*” the separation distance between the plates.

(1) $$C=\frac{\varepsilon_{r}\varepsilon_{0}A}{d}+C_{a}$$

**Figure 3:**
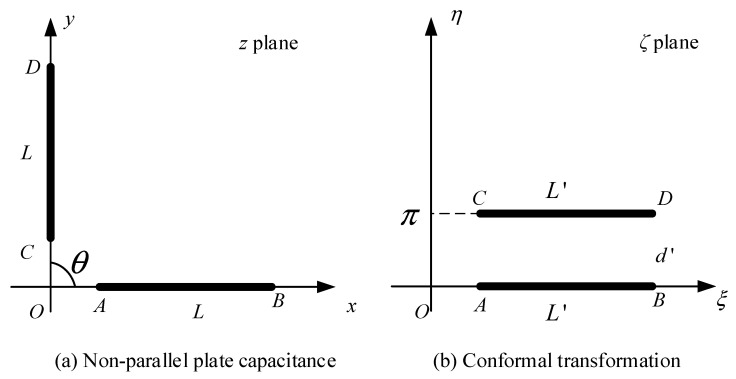
Conformal transformation of non-parallel plate capacitance.

In contrast to the traditional capacitive sensors, the coaxial capacitive sensor network proposed in this paper includes two different types of electrodes: non-parallel plate electrodes and curved plate electrodes. Firstly, two mathematical models are established for analysis, respectively. A pair of non-parallel electrode plates are abstracted from the sensor network and simplified from a three-dimensional space into a two-dimensional space, as shown in Figure 3a. The length and width of the non-parallel plate are set to *l* and *L*, respectively. The plates *AB* and *CD* extend to intersect at point *O*, and the extension line angle is *θ*, where $OA=R_{1}$, $OB=R_{2}$, $L=R_{1}-R_{2}$. According to conformal transformation, a non-parallel plate can be simplified as a pair of parallel plates [19], according to the logarithmic transformation formula,

(2) $$\zeta =\frac{\pi }{\theta }\ln z=\frac{\pi }{\theta }\ln|z|+i\frac{\pi }{\theta }\arg z$$

The area between the plates on the *z* plane can be transformed into a region with width $d^{\prime }$ parallel to the real axis on the ζ plane, as shown in Figure 3b. After the transformation, the width of the *AB* plate is,

(3) $$L^{\prime }=\xi_{A}-\xi_{B}=\frac{\pi }{\theta }\ln\frac{R_{2}}{R_{1}}$$

where $\xi_{A}=\frac{\pi }{\theta }\ln R_{1}$, $\xi_{B}=\frac{\pi }{\theta }\ln R_{2}$, $d^{\prime }=\pi$.

According to the formula of parallel plate capacitance, the non-parallel plate capacitance is calculated as Equation (4).

(4) $$C_{1}=\varepsilon_{0}\varepsilon \frac{S^{\prime }}{d^{\prime }}=\frac{\varepsilon_{0}\varepsilon L^{\prime }l}{d^{\prime }}=\frac{\varepsilon_{0}\varepsilon l}{\theta }\ln\frac{R_{2}}{R_{1}}$$

where $\varepsilon_{0}$ is the vacuum permittivity and ε is the relative permittivity of lubricating oil.

**Figure 4:**
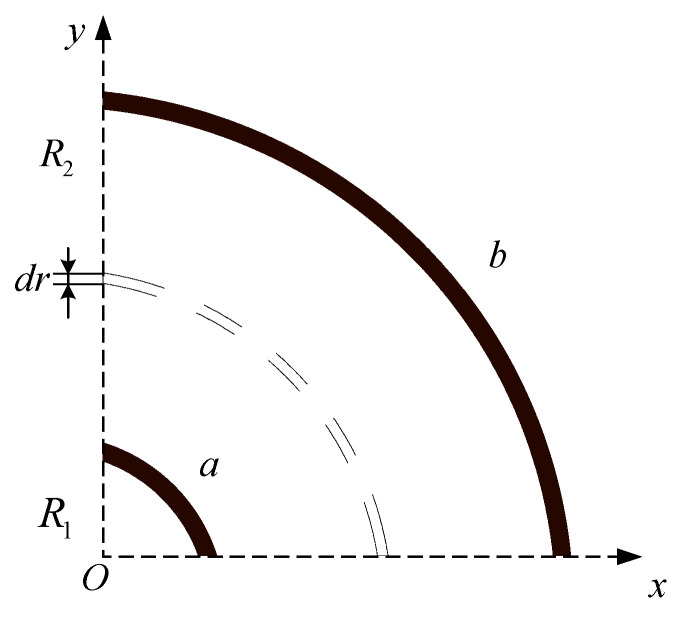
Schematic diagram of curved plate electrode.

Similarly, a pair of curved electrode plates are abstracted from the sensor network, which is simplified into a two-dimensional space, as shown in Figure 4. The radius of the curved plate electrodes (*a* and *b*) is set as $R_{1}$ and $R_{2}$, respectively, and the length of the electrodes is *l*. The edge effect at both ends of the plate can be ignored when $l\gg R_{2}-R_{1}$. The curved plates can be considered a plurality of coaxial thin arc shells, and each thin arc shell can be seen as many parallel plate capacitors with area ΔS connected in parallel. The thickness of the thin arc shell is dr, the side area of the arc surface with radius *r* is $\frac{\pi rl}{2}$ and the capacitance of the thin arc shell with area ΔS is formulated as Equation (5).

(5) $$\Delta C=\frac{\varepsilon_{0}\varepsilon \Delta S}{dr}=\frac{\varepsilon_{0}\varepsilon \pi rl}{2dr}$$

The curved plate electrodes capacitor can be regarded as many coaxial thin arc shell capacitors connected in series, and the capacitance of the curved plate is formulated as Equation (6).

(6) $$\frac{1}{C_{2}}=\int_{R_{1}}^{R_{2}}\frac{2}{\varepsilon_{0}\varepsilon \pi rl}dr=\frac{2}{\varepsilon_{0}\varepsilon \pi l}\ln\frac{R_{2}}{R_{1}}$$

(7) $$C_{2}=\frac{\pi \varepsilon_{0}\varepsilon l}{2\ln\frac{R_{2}}{R_{1}}}$$

where $\varepsilon_{0}$ is the vacuum permittivity and ε is the relative permittivity of lubricating oil.

According to Equation (3), the capacitance of the non-parallel plate is $C_{1}=\frac{\varepsilon_{0}\varepsilon l}{\theta }\ln\frac{R_{2}}{R_{1}}$ when *θ* is taken as $\frac{\pi }{2}$, then

(8) $$C_{1}=\frac{2\varepsilon_{0}\varepsilon l}{\pi }\ln\frac{R_{2}}{R_{1}}$$

According to Equations (6) and (7), we know that the ratio between different types of capacitors in the same subspace is described as Equation (9).

(9) $$\frac{C_{2}}{C_{1}}={\left(\frac{\pi }{2\ln\frac{R_{2}}{R_{1}}}\right)}^{2}$$

It can be seen from the above formula that the ratio between different types of capacitors in the same subspace is related to the radius of the pipeline and the radius of the matrix’s core.

## 3. Numerical Simulation

From the establishment of the mathematical model and the calculation of the capacitance in Section 2, it can be seen that the distance between the oil pipeline and the inner core of the substrate will affect the capacitance of the sensor network when the length of the plate and the permittivity between the plates remain unchanged. In order to better obtain the different parameters of the sensor network, numerical simulations are carried out using the Comsol Multiphysics finite element analysis software. The triangular mesh is applied to numerical calculation. The finer cells are selected to improve calculation precision. The networked sensors are divided into 676,960 domain units. The simulation parameters are listed in Table 1.

### 3.1. Parametric Analysis of Capacitive Sensor Network

**Figure 5:**
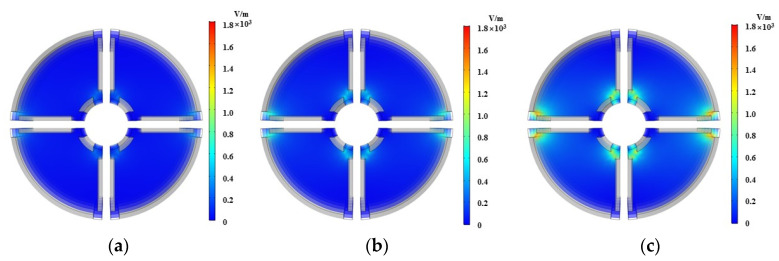
Electric field distribution with different voltages. (**a**) 0.25 V; (**b**) 0.5 V; (**c**) 1 V.

**Figure 6:**
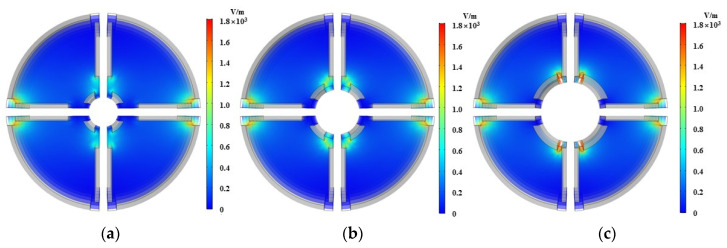
Electric field distribution with different inner core radius. (**a**) 2 mm; (**b**) 3 mm; (**c**) 4 mm.

On the basis of simulation, the distance between the oil pipeline and the core of the substrate ($R_{2}-R_{1}$) and the effect of the applied voltage on the capacitance value are mainly studied. the internal permittivity of the sensor to is set to 80, the oil pipeline radius *r* to 11 mm and the sensor length *l* to 80 mm. One variable remains unchanged and the other is adjusted, respectively. The voltage is set as 0.25, 0.5 and 1 V while keeping $R_{1}=3\;\mathrm{mm}$; the inner core radius $R_{1}$ is set as 2, 3 and 4 mm, respectively, keeping the applied voltage as 1 V. The simulation results are shown in Figure 5 and Figure 6.

**Figure 7:**
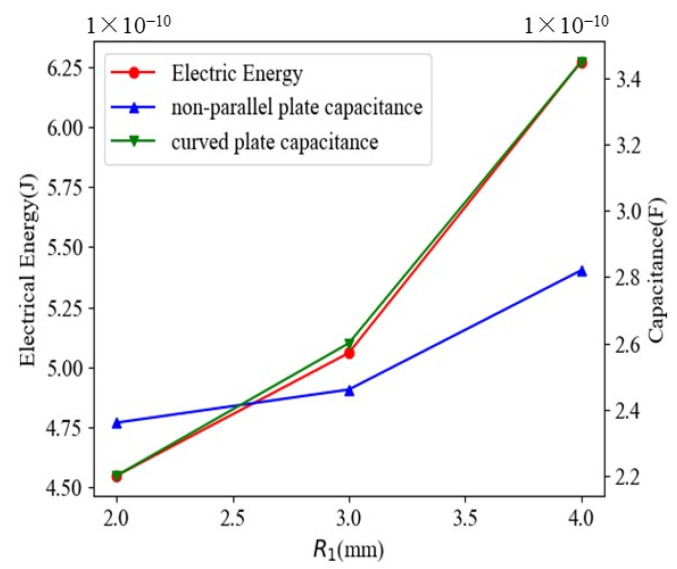
Variation curves of electrical energy and capacitance values with different $R_{1}$ values.

It can be seen from Figure 5 and Figure 6 that the electric field is mainly concentrated on the edge of the plate due to the cross-distribution of the sensor structure. From Figure 5, the electric field strength of the sensor increases with the increase of voltage when the distance between the oil pipeline and the inner core of the matrix remains unchanged. It can be seen from Figure 6 that when the voltage is constant, as the distance between the oil pipeline and the core of the matrix increases, the variation of the electric field strength is small, which is not clearly shown in the figure. It is found that when the distance between the oil pipeline and the core of the matrix is increased, the total electric energy in the sensor increases accordingly, as shown in Figure 7. Meanwhile, the capacitance values of the two different types of plates increase with the increase of the distance between the oil pipeline and the core of the matrix. Considering the influence of the signal-to-noise ratio and flow stagnation, $R_{1}$ is selected as 3 mm; the greater the voltage applied between the electrodes, the greater the electric field strength of the sensor network and the better the signal-to-noise ratio. Therefore, the voltage is selected as 1 V.

**Figure 8:**
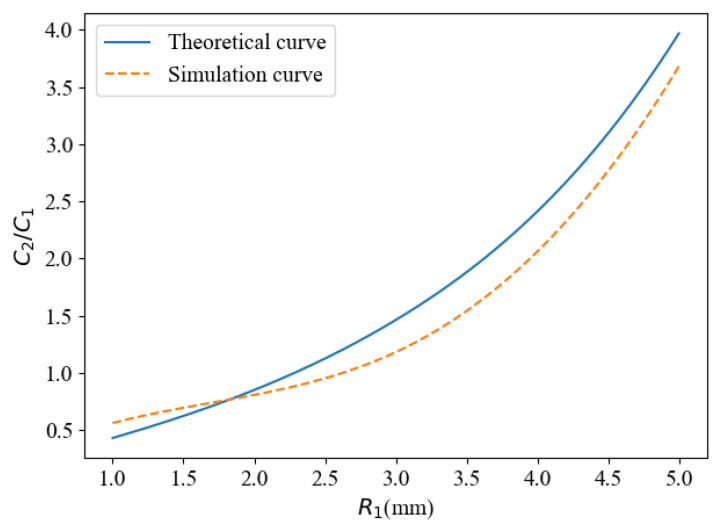
Comparison of theoretical curve and simulation curve.

The capacitance between different types of electrodes is obtained by the global calculation of Comsol Multiphysics, and the ratio between the two capacitors is calculated. Comparing the theoretical value of Equation (8) with simulation data, as shown in Figure 8, it can be seen that the trend of the two curves is basically the same, and the coincidence degree is high. It is indicated that the theoretical analysis and simulation results are very close. Ideally, if the capacitance of one capacitor is known, we can just use the analytical formulation to calculate the capacitance of the other capacitor, especially when two sensors cannot be measured simultaneously due to the limitation of the channel. In practical application, there is a coupling effect that will directly affect signal interpretation when electric fields are applied simultaneously to two pairs of electrodes in the same space.

### 3.2. Numerical Simulation with Debris

**Figure 9:**
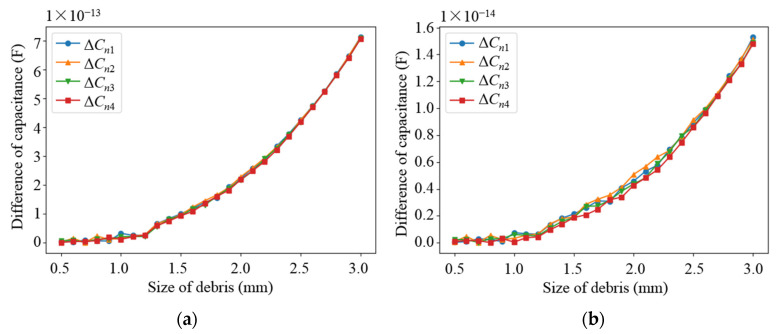
Variation curve of capacitance difference of non-parallel plate with debris size: (**a**) Capacitance variation in water medium; (**b**) Capacitance variation in oil medium.

**Figure 10:**
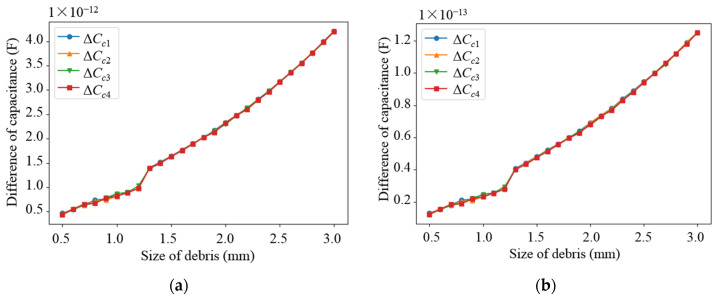
Variation curve of capacitance difference of curved plate with debris size: (**a**) Capacitance variation in water medium; (**b**) Capacitance variation in oil medium.

**Figure 11:**
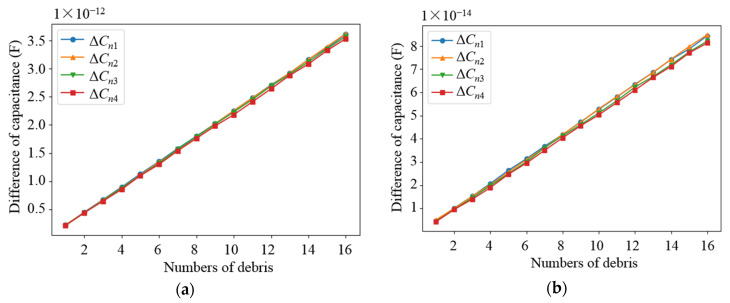
Variation curve of capacitance difference of non-parallel plate with the number of debris: (**a**) Capacitance variation in water medium; (**b**) Capacitance variation in oil medium.

**Figure 12:**
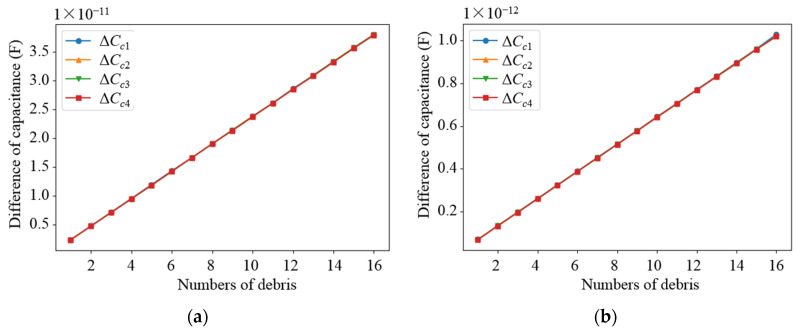
Variation curve of capacitance difference of curved plate with the number of debris: (**a**) Capacitance variation in water medium; (**b**) Capacitance variation in oil medium.

Based on the parameters determined from the above parametric analysis, the sensor network was modeled. The effects of different debris sizes and different debris quantities on the capacitance values of different types of electrodes in the sensor network were simulated, in which the permittivity of the dielectric in the sensor network was set as 80 (water) and 2.2 (lubricating oil), respectively. Debris was added into one of the subspaces when the other subspaces remained unchanged. As shown in Figure 9 and Figure 10, as the diameter of the debris increases, the difference in the capacitance of the two different types of electrode plates shows an increasing trend. It can be observed that as the amount of debris increases, the difference in capacitance grows as well, as shown in Figure 11 and Figure 12. Among them, $\Delta C_{n1}\sim \Delta C_{n4}$ is the difference in the capacitances of the non-parallel plate and $\Delta C_{c1}\sim \Delta C_{c4}$ is the difference in the capacitances of the curved plate.

## 4. Experiments

### 4.1. Experiment Setup

**Figure 13:**
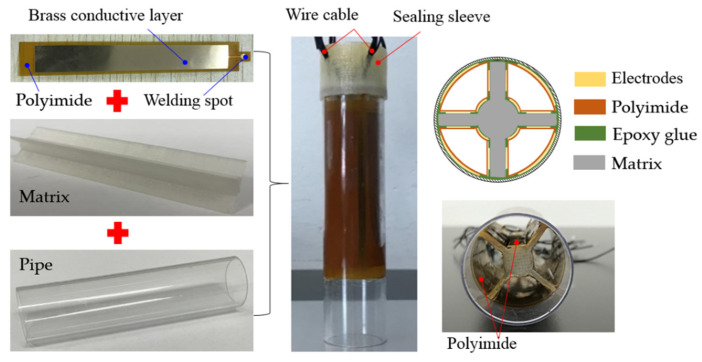
Sensor network based on flexible film electrode.

The following experiments are designed to verify the theoretical derivation and numerical model. According to the simulation results, the length of the coaxial capacitive sensor network is 80 mm, the outer radius of the sensor network is 11 mm, the inner core radius of the matrix is 3 mm and the thickness of the pipe is 1.5 mm. The material used for the electrode plate is a composite flexible polyimide film, where the brass is encapsulated into one side of the flexible film, as shown in the upper left subfigure of Figure 13. To avoid short circuits between electrodes, the side with the brass-conductive layer of the polyimide film is bonded on the pipe or the PLA matrix using the epoxy glue. All the electrodes are separated from the lubricant medium, as well as each other. The packaging process of different components is illustrated in the upper right subfigure of Figure 13. The wire cable of each electrode is welded on the welding spot in order to connect the impedance meter. The connect points of the wire cables are encapsulated by the sealing sleeve, as shown in the middle subfigure of Figure 13. The components of the real developed sensor are shown in Figure 13.

**Figure 14:**
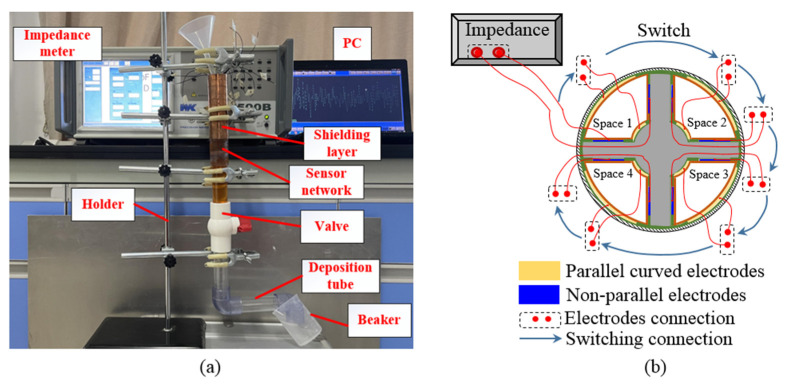
Experimental equipment and test process: (**a**) Illustration of the whole experimental platform; (**b**) Illustration of test process.

In order to improve the convenience of the experimental operation and the accuracy of the monitoring results, an experimental monitoring platform was designed, as shown in Figure 14a, which connects the impedance meter with the sensor network, while a shielding layer is applied to the outside of the sensor network to shield the influence of the external electromagnetic field. The test process of the coaxial capacitive sensor network is illustrated in Figure 14b, where each sensor is measured in turn by switching the electrode connected with the impedance meter. In the way of free fall, abrasive particles pass through the sensor dynamically, and the capacitive signals are measured dynamically by the impedance meter. Although the lubricant in the sensor is stationary, the speed at which the abrasive particles pass through the sensor can be adjusted by the height change of the free-falling body. Actually, the surface of flexible PI film has good insulation and non-wetting properties. Meanwhile, the sensor is vertically placed in the lubrication system. Therefore, the probability of abrasive particles being adsorbed to the sensor surface is very small. Before the monitoring starts, the water valve in the experimental platform is closed, and the intermediate medium (water or lubricating oil) is injected; after the monitoring is finished, the water valve is opened and the liquid mixed with the debris is filtered through the screen and finally flows into the beaker, while the abrasive particles which are deposited in the deposition tube for subsequent collection. The data collected through the impedance meter can be transmitted to the PC and displayed.

### 4.2. Frequency Selection

**Figure 15:**
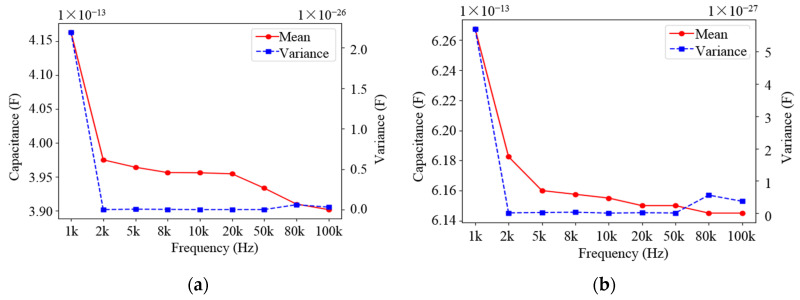
Variation curve of the mean and variance of the non-parallel capacitance with frequency: (**a**) Variation in water medium; (**b**) Variation in oil medium.

**Figure 16:**
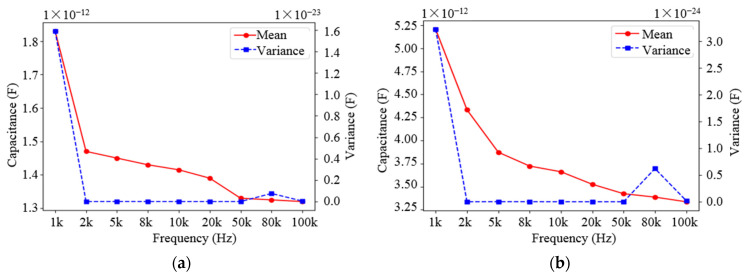
Variation curve of the mean and variance of the curved capacitance with frequency: (**a**) Variation in water medium; (**b**) Variation in oil medium.

In Section 3, the simulation confirmed that the voltage used in the experiments was 1 V, but the frequency of the voltage was not determined. The frequency of the voltage was set to 1 kHz, 2 kHz, 5 kHz, 8 kHz, 10 kHz, 20 kHz, 50 kHz, 80 kHz and 100 kHz, respectively, and the capacitance of the sensor network under different medium was measured when the voltage frequency changed. The curves of the mean and variance of the capacitance changing with frequency are shown in Figure 15 and Figure 16.

From the variation curves in Figure 15 and Figure 16, it can be seen that the values of two different types of capacitances decrease as the voltage frequency increases, regardless of the medium. Meanwhile, it can be seen that when the voltage frequency *f* = 2 kHz, the variance of the obtained capacitance values is the smallest, which means that the capacitance values are more stable; the capacitance values at this frequency are also relatively large. Therefore, the voltage frequency was chosen as 2 kHz in the experiment. It should be noted that the effect of excitation frequency is implicit, and the optimal frequency is determined by trial and error. Generally, when the sensor is tested by the impedance meter, its measured capacitance value changes with the change of excitation frequency due to the existence of the resonant frequency of the sensor itself.

### 4.3. Debris Monitoring

**Figure 17:**
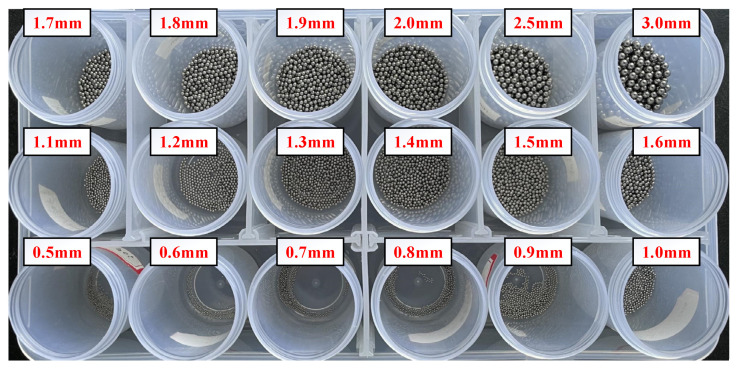
Eighteen types of stainless steel pellets used in the experiment.

**Figure 18:**
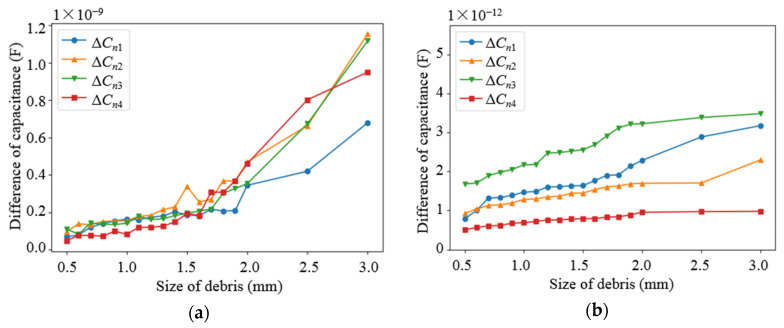
Variation curve of non-parallel plate capacitance with debris size: (**a**) water; (**b**) lubricating oil.

**Figure 19:**
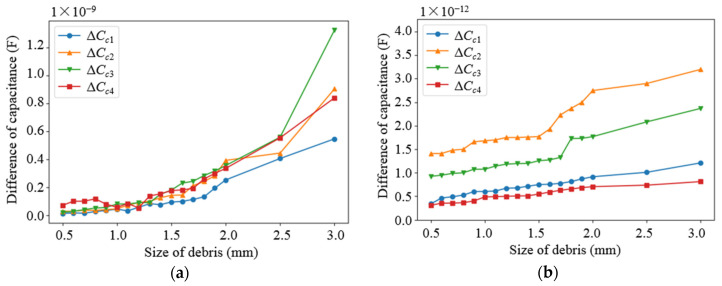
Variation curve of curved plate capacitance with debris size: (**a**) water; (**b**) lubricating oil.

In order to verify the feasibility of the sensor network, metal balls are added to the sensor network to explore the effect of debris on the capacitance value. The sizes of the real debris produced in the engine are from the μm to mm level. For practical purposes, abrasive particles with a maximum size greater than 100 μm should be detected with high accuracy. However, as a conceptual preliminary study and experimental feasible verification, the present study chooses the simulated debris with sizes from 0.5 mm to 3 mm in order to master the basic mechanism and the performance of the proposed coaxial capacitive sensor network. The material of the small balls used in the experiment is 403 stainless steel, as shown in Figure 17. The effect of debris size on capacitance value was explored. Firstly, the metal pellets with diameters of 0.5~3.0 mm were used in the experiment. As shown in Figure 18 and Figure 19, with the increased size of the debris, the value of the capacitance caused by the debris increases, which is in accordance with the results of numerical simulation.

**Figure 20:**
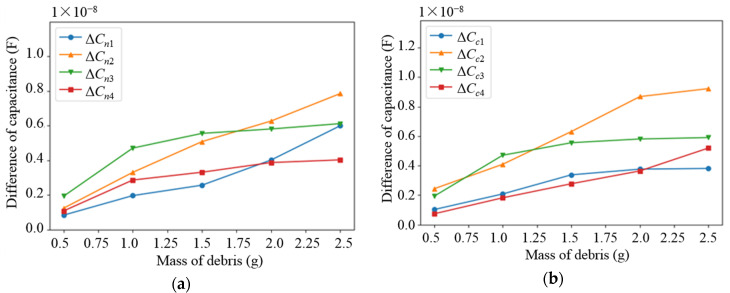
Variation curve of capacitance difference of different types of electrode plates with the mass of debris: (**a**) non-parallel plate; (**b**) curved plate.

The second experiment was used to verify the sensitivity of the sensor network to the mass of metal pellets. The medium (water) was pre-set in the experimental monitoring platform, and 18 types of metal pellets were mixed. The debris clusters with a mass of 0.5 g, 1.0 g, 1.5 g, 2.0 g and 2.5 g were, respectively, placed into the sensor network. The experimental results are shown in Figure 20. It can be seen that the two different types of capacitances in the subspaces increase as the mass of debris increases. Meanwhile, the experimental results correspond to the results of the numerical simulation. In summary, the feasibility of the sensor network is illustrated by the linear relationship exhibited on the different sizes and masses of debris.

## 5. Conclusions

As an important part of aero-engine health management, oil debris monitoring plays an important role in the safety assessment of engine systems. This paper proposed a coaxial capacitive sensor network, which can be integrated into the oil pipeline for online monitoring. The flow space of the sensor network was divided into multiple spaces. When the wear debris passed through any of these spaces, the dielectric constant between the electrodes and the capacitance between the electrodes was changed accordingly. Meanwhile, the mathematical models of different types capacitors (including non-parallel plate capacitors and curved plate capacitors) were established and the relationship between the two kinds of capacitors was analyzed theoretically. In addition, the mathematical model was verified by numerical simulation, and the optimal parameters of the sensor network were obtained. Based on the obtained parameters, a sensor network based on a flexible film electrode was fabricated. The feasibility of the sensor network was verified by performing experiments with the addition of debris in different media (water and lubricant oil). The experimental results show that the capacitance value increases almost linearly with the increase in the size and mass of abrasive particles. However, the experiments in this paper are only preliminary verification. Further work will focus on the characterization of the capacitance to debris measured at different types of electrodes, as well as the improvement of the measurement accuracy and the influence of several factors on monitoring results.

## Figures and Tables

**Table 1:** Parameters of simulation experiments.

Name	Value	Describe
*ε*_0_	8.854187817 × 10^−12^ F/m	Vacuum dielectric constant
*ε*	Variable	Dielectric relative permittivity
*l*	80 mm	Sensor network length
*R*_1_	Parameters to be determined	Inner core radius
*R*_2_	11 mm	Sensor network radius
*U*	Parameters to be determined	Excitation plate voltage
